# Metabolomics Analysis Uncovers Distinct Profiles of Liver Post-Transplant Patients by Immunosuppression Regimen

**DOI:** 10.3390/metabo15110700

**Published:** 2025-10-29

**Authors:** Cristina Baciu, Bima J. Hasjim, Saba Maleki, Elisa Pasini, Meera Kennedybhai Patel, Maryam Shojaee, Amirhossein Azhie, Giovanna Saracino, Sumeet K. Asrani, Mamatha Bhat

**Affiliations:** 1 Ajmera Transplant Program, University Health Network, Toronto, ON M5G 2N2, Canada; kristinabaciu@hotmail.com (C.B.); saba.maleki@uhn.ca (S.M.); elisa.pasini@uhn.ca (E.P.); mpatel12027@meds.uwo.ca (M.K.P.); 2 Department of Surgery, School of Medicine, UC Irvine, Irvine, CA 92697, USA; bhasjim@hs.uci.edu; 3 Department of Internal Medicine, Rady Faculty of Health Sciences, University of Manitoba, Winnipeg, MB R3T 2N2, Canada; shojaeem@myumanitoba.ca (M.S.); azhiea@myumanitoba.ca (A.A.); 4 Baylor Scott & White Health, Dallas, TX 75226, USA; giovanna.saracino@bswhealth.org (G.S.); sumeet.asrani@bswhealth.org (S.K.A.)

**Keywords:** metabolomics, post-transplant complications, lipid metabolism, immunosuppression treatment

## Abstract

Background/Objectives: Long-term survival among liver transplant (LT) recipients who live beyond one year has remained relatively stable over recent decades. However, reducing long-term morbidity is increasingly important, and metabolomics may enable risk-based, personalized immunosuppression. We aimed to evaluate and compare the serum metabolomic profiles of LT recipients treated with tacrolimus (TAC) versus sirolimus (SIR), to elucidate metabolic pathways associated with these regimens. Methods: Targeted metabolomic profiling of 894 metabolites was conducted on serum samples from 128 LT recipients using the Biocrates MxP^®^ Quant 500 kit. Data were analyzed with MetaboAnalyst 6.0, and multivariate analysis was performed using Partial Least Squares-Discriminant Analysis (PLS-DA). Metabolites with Variable Importance in Projection (VIP) scores > 1.5 underwent pathway enrichment in OmicsNet, incorporating Gene Ontology annotations and Kyoto Encyclopedia of Genes and Genomes (KEGG)-based network analysis. Results: Eighty-seven metabolites were significantly altered between groups. Phosphatidylcholines (PCs) and ceramides were elevated in TAC-treated patients, while di- and triacylglycerols were higher in the SIR group. Pathway enrichment implicated lipid metabolism, particularly glycerophospholipid, ether lipid, and sphingolipid pathways. Network analysis identified enriched modules related to metabolic regulation and immune response. Conclusions: Divergent metabolomic profiles distinguish TAC- and SIR-treated recipients, suggesting regimen-specific impacts on lipid metabolism with potential relevance to post-transplant complications.

## 1. Introduction

Although short-term survival after liver transplantation (LT) has improved over time through medical and surgical advancements, long-term survival has plateaued in contrast. Among LT patients who have survived at least 1 year post-LT, long-term post-LT survival has been similar over the past 30 years [1]. However, there is growing recognition of the need to reduce long-term morbidities, such as cardiovascular disease. Metabolomics offers an opportunity to stratify patients by risk, enabling more tailored immunosuppression strategies that address individual vulnerability and improve overall health beyond survival alone.

Through advancements in immunosuppression strategies, rejection leading to graft failure and death are now relatively rare and account for only 1.7% of all long-term deaths. However, long-term sequelae of immunosuppression have emerged as common causes of late death among transplant recipients [1,2]. Thus, there is still room to optimize immunosuppression strategies to mitigate these long-term risks [3,4]. The immunosuppressive drugs can change metabolic profiles.

Our prior review highlighted the impact of these alterations on transplant survival and patient prognosis [5,6]. Serum metabolites may provide some insights to capture subtle differences between immunosuppression strategies among transplant candidates and recipients [7].

In the pre-LT setting, unique metabolomic profiles have been identified among metabolic dysfunction (e.g., insulin resistance, lipid metabolism) associated with liver fibrosis and its progression towards cirrhosis [8,9]. In the post-LT setting, differences in metabolomic profiles may indicate recurrent or new-onset post-transplant metabolic syndromes [10,11,12].

Furthermore, there is emerging evidence that emphasizes that different immunosuppression drugs may lead to different metabolomic profiles (e.g., short-chain fatty acids (SCFAs), amino acids (AAs), and bile acids (BAs)) that uniquely interact with host cell receptors [13,14,15,16]. Lastly, metabolic biomarkers may give rise to earlier detection of these insidious long-term complications, and precisely identifying such biomarkers may spur earlier intervention before the onset of systemic progression.

Hypothesis: post-LT patients maintained on tacrolimus versus sirolimus exhibit distinct serum metabolomic profiles, which can provide mechanistic insights and enable a more personalized approach to immunosuppression management.

## 2. Materials and Methods

### 2.1. Study Design and Participants

In this cross-sectional observational study, serum samples were retrieved from consented recipients who underwent LT between January 2010 to March 2016 from Baylor Scott & White Health (BSWH), TX, USA. The study comprised 128 post-LT patients, who were classified based on their immunosuppressive medication type, e.g., tacrolimus (TAC) or sirolimus (SIR), and time since transplantation (one or two years). Most samples were collected over a one-year period (36 SIR and 72 TAC), with the exception of eight SIR and 12 TAC samples collected over a two-year time frame.

Inclusion criteria: Adult liver transplant recipients, who underwent liver transplants between 2010 and 2016 at the Baylor Simmons Transplant Institute in Dallas with stored serum samples in the institutional biobank, were included. Exclusion criteria: Patients were excluded if they had received prednisone for more than three months, had renal dysfunction with a glomerular filtration rate of less than 60 mL/min; had an active infection; biliary or vascular complications; steatohepatitis; history of previous transplant; or multiorgan transplantation.

Sample Collection: Peripheral venous blood samples were collected from participants using standard aseptic technique. Blood was drawn into BD Vacutainer^®^ SST™ serum separation tubes (BD catalog #367985), which contain a clot activator and gel for serum separation. After collection, the tubes were allowed to clot upright at room temperature for 30 min. Clotted samples were centrifuged at 2000× *g* for 10 min at 4 °C to isolate the serum. The separated serum was aliquoted into pre-labeled cryovials and immediately stored at −80 °C until analysis.

Metabolomics Analysis: Targeted metabolomic profiling was performed using the Biocrates MxP^®^ Quant 500 kit on 128 serum samples at the SPARC BioCentre, Hospital for Sick Children, Toronto, Canada. The Biocrates metabolomics technology, more specifically, the MxP^®^ Quant 500 kit [17]. This specialized assay enabled the accurate identification and quantification of 894 native and combinations of metabolites encompassing amino acids, acyl carnitines, biogenic amines and their derivatives, uremic toxins, glycerophospholipids, sphingolipids, hexoses (including glucose), as well as fractions or sums of specific features. For quality control, a system suitability test (SST) was performed prior to each experiment. Experiments were initiated only if the SST passed in the Biocrates analysis software (WebIDQ). For the SST, the blank LC sample consisted of 50% methanol, while the test LC sample was a human plasma-based sample containing internal standards and all metabolites measured in the LC component of the analysis. For FIA, the blank consisted of the FIA solvent provided in the Biocrates kit, and the test FIA sample contained typical metabolites of the FIA component. Internal standards were pre-spotted on the kit plate filters by Biocrates and were not added manually.

Analytical performance was rigorously evaluated using Biocrates-provided calibration and validation quality control samples spanning three defined concentration levels. Expected reference values for each level were supplied by Biocrates in accordance with the corresponding kit lot number specifications. Blank or zero samples were prepared by spotting nothing or 10 µL of PBS onto the well filter, respectively, followed by standard plate processing according to Biocrates’ instructions.

All measurements were performed on a Sciex QTRAP 6500+ mass spectrometer coupled to an Exion LC system.

### 2.2. Data Processing and Analytical Methods

Data analysis: Metabolite concentrations, expressed in µM (absolute concentrations) for each sample, were input into the MetaboAnalyst 6.0 software [18] for bioinformatics analysis. For the two-group comparison, data processing included: (i) excluding features with over 50% missing data or those with constant values across samples, (ii) imputing missing values by replacing them with 1/5 of the minimum positive value, and (iii) normalizing through quantile normalization, log10 transformation, and autoscaling. Multivariate analysis was subsequently conducted using Partial Least Squares—Discriminant Analysis (PLS-DA) to identify significant metabolites, based on Variable Importance in Projection (VIP) scores calculated for the first component. Metabolites with VIP > 1.5 were considered significant and were categorized into compound classes according to the Human Metabolome Database (HMDB) and Biocrates’ documentation, especially for non-standard features such as fractions and sums.

Graphical representation: The normalized levels of selected metabolites in SIR- and TAC-treated groups were compared and plotted using GraphPad Prism version 10.4.2. Statistical *p*-value was computed by the Mann–Whitney test, and the violin plots were presented with median (continuous line) and 95% confidence intervals (dashed lines).

Network and pathway enrichment analysis: Selected metabolites with HMDB identifiers, together with their fold change, were further used for pathway enrichment analysis using OmicsNet [19]. This software employs various metabolite-protein interaction databases, and we opted for the Kyoto Encyclopedia of Genes and Genomes (KEGG) reactions. We also analyzed the largest subnetwork generated using Module Explorer. This approach analyzes the network by identifying tightly connected subnetworks or modules. The InfoMap algorithm reveals community structures by compressing information flow representations through random walks [20]. For module functional annotation, we selected several Gene Ontology (GO) terms included in the software: ‘GO:BP’ (Gene Ontology/Biological Process), ‘GO:CC’ (Gene Ontology/Cellular Component), and ‘GO:MF’ (Gene Ontology/Molecular Function).

Statistical analysis: All statistical analyses were carried out using R (version 4.4.3; R Core Team, 2025) and GraphPad Prism (version 10.4.2; GraphPad Software, LLC). For the clinical characteristics summarized in Table 1, categorical variables were compared using either Fisher’s exact test or the chi-squared (χ^2^) test, as appropriate. Continuous variables were assessed using the Kruskal–Wallis rank sum test. Additional details regarding statistical methods applied to other analyses are provided in the relevant sections.

## 3. Results

### 3.1. Study Population and Clinical Characteristics

A total of 128 post-LT participants were enrolled in the study, of whom 44 (34.4%) received SIR, while 84 (65.6%) received TAC. Demographic data and clinical characteristics are presented in Table 1. Patients in the TAC group were younger (51.0 ± 11.3 years vs. 56.2 ± 9.4 years, *p* = 0.004) than those in the SIR group, although other patient and donor characteristics (e.g., sex, indication for transplant, comorbidities) were comparable between the two groups. Patients receiving TAC and SIR had similar Model for End-Stage Liver Disease (MELD) scores and other serum biomarkers (e.g., creatinine, glucose, Guanosine-5′-triphosphate (GTP), Alanine Aminotransferase (ALT), alkaline phosphatase (ALP), gamma-glutamyl transferase (GGT)). Graft survival rates were comparable between treatment arms (Supplementary Figure S1).

### 3.2. Metabolomic Data Processing and Multivariate Analysis

**Figure 1:**
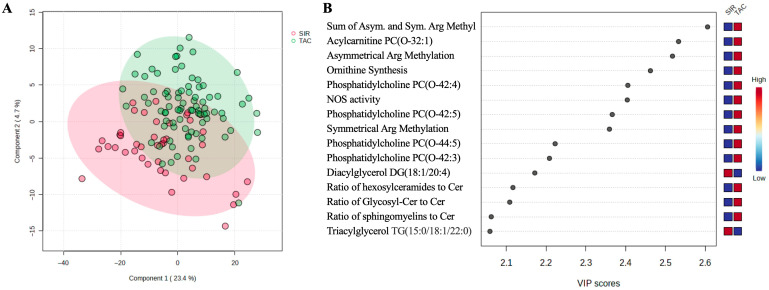
Multivariate analysis showing: (**A**) PLS-DA plot in 2D; (**B**) top 15 features (VIP > 2). For the full names please of the abbreviated terms in this panel, please refer to Supplementary Table S1.

**Figure 2:**
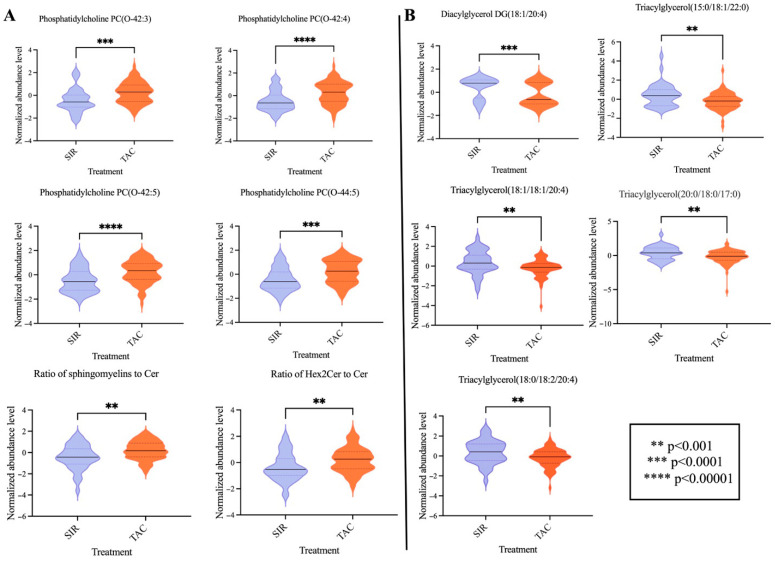
Violin plots of significantly altered metabolites (median with 95% CI) illustrating the levels of specific: (**A**) phosphatidyl cholines and ceramides; and (**B**) di- and tri-acylglycerols by the immunosuppression regimen. Statistical *p*-value was computed with the Mann–Whitney non-parametric test.

From the total of 894 features (metabolites and some combinations), 47 with a constant or single value across the samples were deleted, while 18.9% of missing values were replaced by one-fifth of the minimum positive value, as explained in the Materials and Methods section. Following these steps, normalization was applied before proceeding further with PLS-DA analysis. A fair separation by the treatment group can be observed from the PLS-DA plot, with 23.4% of the variance being represented by the first component (Figure 1A). Five-fold cross-validation results of the PLS-DA model, including accuracy, R2, and Q2, are shown in the Supplementary Table S1. Our analysis identified 87 significantly changed features with VIP score > 1.5 (Supplementary Table S2), of which the top 15 is plotted in Figure 1B. Six of the top discriminatory features are represented by phosphatidylcholines, all of which are upregulated in patients treated with TAC. Also, a couple of ceramides and the ratio of these are upregulated in the TAC-treated patients (Figure 2A). In contrast, several di- and tri-acylglycerols (or triglycerides) exhibited significantly higher levels in SIR treated group (Figure 2B).

### 3.3. Network and Pathway Enrichment Analysis

**Figure 3:**
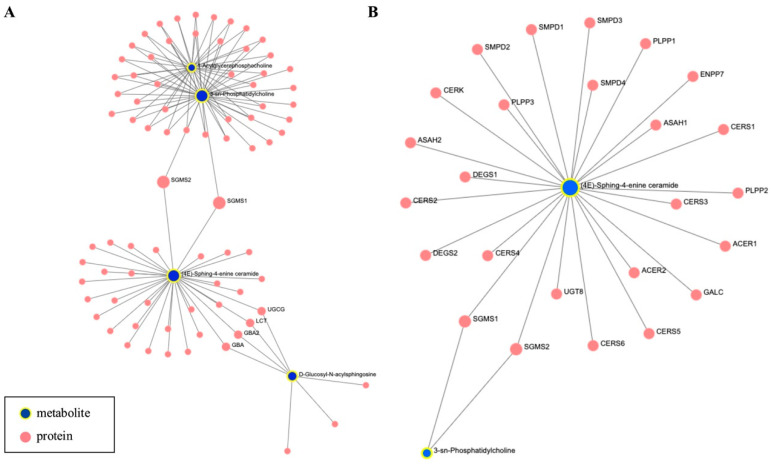
Network analysis. (**A**) The largest subnetwork built by OmicsNet; (**B**) molecules from this subnetwork that belong to the glycerophospholipid metabolism.

Our pairwise analysis, SIR- vs. TAC-treated groups, revealed 87 significantly altered features. Of these, 47 were individual metabolites with HMDB symbols that were used, together with their corresponding fold changes, as input for network and enrichment analysis with OmicsNet [19]. In building the network, we opted to add mRNA proteins from the KEGG metabolite-protein database, curated by the software. The largest subnetwork built (subnetwork 1) consists of 156 nodes, 199 edges, and 12 seeds, of which four were represented by metabolites from our list, such as acyl-glycerophosphocholine, phosphatidylcholine, sphing-4-enine-ceramides, and D-glucosyl-N-acylsphingosine, as presented in Figure 3A. Pathway enrichment analysis with all the nodes in the network identified several lipid metabolism pathways, of which the glycerophospholipid metabolism was the most significantly altered (FDR = 1.12 × 10^−70^), followed by the ether lipid metabolism (FDR = 8.65 × 10^−60^) and the sphingolipid metabolism (FDR = 1.08 × 10^−49^). The complete list of top 10 enriched pathways is presented in Table 2, with metabolites specifically involved in glycerophospholipid metabolism detailed in Figure 3B.

### 3.4. Functional Module Analysis

**Figure 4:**
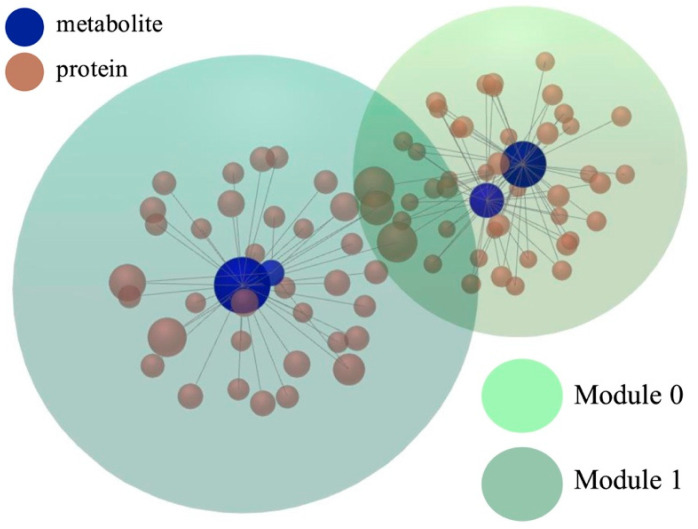
Two major modules were identified using the InfoMap algorithm. Module 0 has 44 nodes and a *p*-value of 3.72 × 10^−18^; Module 1 contains 31 molecules and has a *p*-value of 1.63 × 10^−11^. Functional annotation of the two modules is detailed in Table 3.

Module Explorer analysis using the InfoMap algorithm [20] identified two distinct functional modules within subnetwork 1 (Figure 4). Module 0 has 44 nodes and a *p*-value of 3.72 × 10^−18^, whereas Module 1 contains 31 molecules and has a *p*-value of 1.63 × 10^−11^. Further functional analysis and annotation with GO terms such as biological process (BP), cellular component (CC), and molecular function (MF) categories revealed substantial functional overlap between modules, with Module 0 showing greater enrichment depth. The top functional annotations for both modules are summarized in Table 3.

## 4. Discussion

This study investigated the metabolomic profiles of liver recipients treated with TAC and SIR. Although similar clinical outcomes were observed, we found that phosphatidylcholine metabolites and ceramides were upregulated in LT recipients on TAC. Additionally, acylglycerols were specifically elevated in those receiving SIR. Furthermore, pathway enrichment analyses identified that lipid metabolism pathways were particularly involved, with glycerophospholipid metabolism being the most significant, followed by ether lipid metabolism and sphingolipid metabolism. Metabolomics reflects the downstream products of cellular activity and probably reflects the earliest cellular responses to pharmacological stress [21], and could therefore be useful for understanding immunosuppressive effects and predicting toxicity patterns.

Understanding the different toxicity patterns and immunosuppressive mechanisms of TAC, as a calcineurin inhibitor, and SIR, as an mTOR inhibitor, helps in appreciating our metabolomic data. Given that TAC has potent anti-rejection properties, it is usually utilized to decrease T-cell activation by inhibiting calcineurin, which consequently inhibits NFAT (nuclear factor of activated T cells) signaling. Meanwhile, the usage of TAC can be restricted due to neurotoxicity, dyslipidemia, new-onset diabetes, HTN, and nephrotoxicity risks. Among LT recipients, TAC has been associated with common metabolic complications, including DM, HTN, and hypercholesterolemia [22,23,24,25]. Despite sirolimus inhibiting mTOR, specifically mTORC1, and binding FKBP-12 to block lymphocyte proliferation downstream of calcineurin. It may potentially protect renal function, as it is not a calcineurin inhibitor. Edema, proteinuria, mouth ulcers, hypertriglyceridemia, dyslipidemia, and hypercholesterolemia are a number of the associated adverse effects, though [26,27,28,29]. Therefore, identifying metabolomic differences between TAC and SIR provides mechanistic insights that may help explain their disparate side-effect profiles and more precisely tailor immunosuppressive strategies, as these two medications have distinct effects on lipid, glucose, and renal physiology.

Moreover, the observed metabolomic differences may also be influenced by patient-related factors. Compared to the SIR group (56.2 ± 9.4 years, *p* = 0.004), the TAC group’s patients were younger (51.0 ± 11.3 years) in our cohort. Age-related factors can impact the pharmacokinetics of TAC and SIR in LT recipients due to decreased hepatic and renal clearance, altered cytochrome P450 activity, and changes in body composition. Therefore, in elderly recipients, higher dose-adjusted medication concentrations and raised susceptibility to side effects, such as dyslipidemia and nephrotoxicity, may be seen as the results of these changes [30]. Although TAC clearance variability depends on age, CYP3A5 genotype, and hematocrit, hematocrit levels should be taken into account in SIR dosage for age-related pharmacokinetic differences [31,32].

Significant clinical implications result from TAC-treated individuals’ increased PC levels. PC is a critical component phospholipid of hepatic cell membranes and serves as a precursor of triacylglycerol and diacylglycerol (DAG) in the liver [33,34]. Decreased PC content can lead to impaired secretion of very low-density lipoprotein secretion [35] and PC deficiency has been linked to Metabolic Dysfunction-Associated Steatotic Liver Disease (MASLD) [36], reduced hepatic regeneration [37], and early graft dysfunction [38]. Our finding that PC is upregulated in TAC-treated patients, while acylglycerols are decreased, suggests a shift toward phospholipid synthesis at the expense of neutral lipid production. Moreover, it is possible that this metabolic pattern adds to the cardiovascular risk profile linked to calcineurin inhibitor treatment. Immunosuppressive effects on endothelial function are one of the many factors contributing to transplant recipients' elevated cardiovascular risk [39,40]. Our findings provide a molecular explanation for the dyslipidemia typically seen with calcineurin inhibitor medication, and they suggest that phospholipid profiling could be used as an early diagnostic for cardiovascular problems [39,40].

These findings support Quinn et al.’s observations in mouse experiments, who found that mTOR complex 1 (mTORC1) played an essential role in lipid homeostasis and regulating PC synthesis [35]. Inhibition of mTORC1 by SIR leads to lipid accumulation in hepatocytes and reduced hepatic triacylglycerol synthesis, which may contribute to steatosis. Meanwhile, increasing PC synthesis in SIR-treated mice was able to reverse hepatosteatosis and restore triglycerides (TAG) secretion [35]. The data indicate that mTORC1 plays a significant role in regulating phospholipid biosynthesis. Clinically, this underscores the importance of supplementing Phosal 50 PG^®^—which contains phosphatidylcholine, propylene glycol, mono and diglycerides, ethanol, soy fatty acids, and ascorbyl palmitate—in the formulation of sirolimus oral tablets [41]. Therefore, PC supplementation in SIR formulations is supported by this molecular insight, which also raises the possibility that the PC profile could be used as an early diagnostic for cardiovascular problems.

Gosis et al. found that selective modulation of hepatic mTORC1 signaling through genetic deletion of Flcn and activation of transcription factor TFE3 affords selective inhibition of mTORC1 and protection against MASLD, respectively [42].

We found that patients treated with SIR had lower ceramide levels. Graft function monitoring is directly impacted by the differences in ceramide levels between treatment groups [43,44]. Ceramides contribute to the synthesis of complex sphingolipids, including glucosylceramide, which plays a vital role in cell growth and receptor-mediated signal transduction [45,46]. Low serum levels of ceramides have also been associated with liver graft dysfunction [47] and acute rejection [48]. In a study of kidney transplant recipients, Burghelea et al. demonstrated that those with high TAC levels (>8 ng/mL) had increased ceramide values (ceramide t18:0/22:0 (2OH)) compared to recipients with lower TAC levels (5 ng/mL) [49]. Similarly, Mucke et al. found that ceramide levels varied with tacrolimus trough levels in a cohort of 149 LT recipients and proposed that pro-apoptotic metabolic states linked to allograft problems could be represented by reduced ceramide levels [47]. In our study, the larger sample sizes of sirolimus-treated patients provide unique insights into their metabolomic profiles compared to the TAC-treated subgroups of the transplant literature. Serum ceramides may serve as a useful biomarker to predict long-term allograft dysfunction, and their downregulation by SIR could potentially be utilized among patients who are particularly at risk. Since short-chain ceramides (like C16) [50,51] have pro-apoptotic properties, but long-chain ceramides (≥C24) [52,53] and their dihydroceramide precursors mostly promote cell growth [54]. As Mucke et al. demonstrated that elevated levels of sphingolipids and serum short-chain ceramides may indicate immunological tolerance, inflammation, and allograft dysfunction after Orthotopic Liver Transplantation (OLT) [47].

Moreover, the combination therapy of a functional sphingosine-1-phosphate (S1P) inhibitor, S1P-analogon fingolimod (FTY720), with TAC can reduce cellular ceramide levels, reduce lymphocyte infiltration, and ultimately lower TAC doses to potentially mitigate the long-term metabolic side effects in murine models [55]. Continued investigations to further delineate the differences in metabolomic profiles among immunosuppression medications, beyond TAC and SIR, will help facilitate a more personalized approach towards immunosuppression management.

Taken together, the lipid metabolism pathway remains active in transplant recipients. Dysfunction in these pathways may lead to hyperlipidemia, which develops in up to 45% of all LT recipients regardless of immunosuppression and is a significant risk factor for major adverse cardiovascular events [56]. Lipid metabolism is a complex process, and each immunosuppression strategy may contribute both protective and exacerbating effects towards dyslipidemia. For example, TAC may mitigate the increase in serum low-density lipoprotein cholesterol (LDL) seen with steroids or cyclosporine, but has been associated with hyperinsulinemia and hypertriglyceridemia [57]. Conversely, SIR may increase hypertriglyceridemia but could mitigate dyslipidemia by enhancing renal recovery and reducing calcineurin inhibitor concentrations. Current guidelines recommend avoiding SIR in patients with atherosclerosis, triglycerides > 500 mg/dL, or LDL-C > 250 mg/dL [57,58].

Despite a possible lower risk of MASLD due to increased phosphatidylcholine and decreased acylglycerols, tacrolimus increases the risk of high cholesterol, diabetes, and hypertension, as well as a more pronounced cardiovascular risk profile, primarily through ceramide-mediated pathways. Compared to sirolimus, which more potently encourages hypertriglyceridemia and hepatic steatosis, these hazards are different [59,60,61].

Shifting clinical endpoints from composite serum biomarkers, such as triacylglycerols and cholesterol, to specific metabolites may enable physicians to support more precise and individualized approaches for managing immunosuppression.

In addition to pathway-level distinctions, our functional annotation analysis revealed enrichment of specific biological processes and molecular functions underlying the observed metabolomic divergence. As shown by our functional annotations, TAC-treated patients exhibited enrichment in glycerophospholipid biosynthetic process, phospholipid metabolic process, and cellular biogenic amine metabolism, reinforcing the central role of phospholipid biosynthesis in tacrolimus-associated profiles. These findings align with studies showing that calcineurin inhibitors upregulate enzymes involved in phospholipid remodeling, such as Phosphatidylethanolamine N-methyltransferase (PEMT) and choline kinase α [33,37]. Also, TAC-treated patients showed enrichment in sphingolipid metabolic, ceramide biosynthetic, and sphingolipid-related pathways, supporting evidence that mTOR inhibition alters sphingolipid metabolism and may promote inflammation [42,62,63].

At the molecular level, enhanced phospholipase A2, lipase activity, and N-acyltransferase activity indicate increased lipid hydrolysis and remodeling. These activities influence membrane fluidity, receptor function, and insulin sensitivity [64,65]. Many were localized to the endoplasmic reticulum, a critical site for lipid processing in hepatocytes, where immunosuppressants may modulate ER stress and lipid droplet formation [34,53]. Taken together, these findings suggest that the metabolic effects of immunosuppression extend beyond gross lipid levels and involve nuanced subcellular lipid regulation, with important implications for post-transplant diabetes mellitus, graft function, and cardiovascular risk [8,57].

Although 5-year outcomes are relatively similar between the two groups, differences in metabolomic profiles across immunosuppression strategies among transplant patients may yield valuable clinical insights towards longer-term outcomes beyond 5 years, though future research is required [8].

### Limitations

Our study should be interpreted within the context of its limitations. The metabolomic data of healthy controls will help in interpreting the results of this study, which is one of the key limitations. Although further studies are highly recommended for determining if these observed metabolite differences, including decreased phosphatidylcholine concentrations in recipients who were treated with SIR compared with those treated with TAC, can be the result of drug-specific effects, the transplantation procedure, or even deviations from normal physiology regarding the lack of baseline values from a healthy control group. This limitation was also supported by previous studies. In Kim et al. [7], cyclosporine A- and TAC-based regimens increased lipid metabolites after kidney transplantation; however, the lack of healthy control data restricted in determine whether these alterations occurred due to normalization or pathology. Similarly, the presence of TAC-associated metabolites in LT recipients was demonstrated in Zhu et al.’s study, despite the absence of healthy control data being mentioned as a limitation [66]. Additionally, we noted that our study’s small sample size limited the generalizability of our findings and likely increased the risk of overfitting in our statistical models. While we proposed some factors that may contribute to this limitation, we recommend that additional studies with larger, multicenter cohorts be conducted to validate these metabolomic signatures.

In addition, the serum samples we collected from our patients for metabolomic analysis were obtained only once. Although we were able to elucidate metabolomic differences between various immunosuppression strategies, these serum samples were collected 1–2 years after liver transplantation (LT). Conducting longitudinal analyses over fixed time points in the post-LT follow-up may yield important insights into the cumulative effect of immunosuppression exposure on these metabolomic profiles. Furthermore, comparing these observations to pre-LT states would be important in future research to minimize the effect of potential confounders. Next, there is limited data on donor characteristics. This information may influence our observations since the chimeric post-LT environment is important to characterize [67,68]. Moreover, food intake and lifestyle factors can affect metabolomics profiles; however, these factors were not possible to be assessed in our study and may represent potential confounders. Lastly, we do not have detailed information on the serum or target trough levels of immunosuppression for each patient. Immunosuppression trough levels have been shown to influence specific metabolite concentrations, which can further yield insights towards allograft outcomes [47]. Ultimately, once the predictive capacity of these metabolomic profiles has been clarified in future studies, these data may assist in guiding clinicians in making informed decisions regarding daily or outpatient immunosuppression management.

## 5. Conclusions

In conclusion, this study evaluated the metabolomic profiles of liver transplant recipients treated with tacrolimus versus those treated with sirolimus. Despite identical 5-year clinical outcomes, different metabolic signatures were identified. Although sirolimus has been linked to elevated di- and tri-acylglycerols and altered lipid metabolic pathways, tacrolimus treatment could lead to up-regulation of phosphatidylcholine and ceramides. These findings reveal that immunosuppressive agents have varied metabolic effects, suggesting that metabolomic profiling may be helpful in tailoring immunosuppression therapy following liver transplant.

## Figures and Tables

**Table 1:** Liver transplant (LT) recipient clinical and laboratory characteristics. Numerical data is presented with median and interquartile (IQR) intervals. N represents the number of patients for whom the information was recorded.

Characteristic	N	SIR, N = 44 ^1^	TAC, N = 84 ^1^	*p*-Value ^2^
**RECIPIENT**				
Age at LT (years)	128	57.50 (53.00, 62.00)	52.00 (47.00, 59.25)	0.004
Sex	128			0.97
Female		14 (32%)	27 (32%)	
Laboratory readings				
ALT (U/L)	128	60.00 (30.50, 198.00)	94.00 (39.00, 457.75)	0.24
AST (U/L)	128	81.50 (53.00, 381.00)	136.50 (63.50, 646.75)	0.23
ALP (U/L)	128	115.50 (93.00, 163.50)	128.50 (88.50, 181.75)	0.54
Creatinine (mg/dL)	126	1.40 (1.00, 1.83)	1.23 (0.80, 1.70)	0.24
MELD at transplant	125	21.50 (13.00, 29.25)	23.00 (17.00, 28.00)	0.44
MELD Na	125	24.10 (14.80, 29.95)	24.40 (18.50, 30.70)	0.49
Primary diagnosis	125			0.12
Acute Hepatic Necrosis		0 (0%)	6 (7.3%)	
Alcohol-related		13 (30%)	25 (30%)	
Autoimmune		2 (4.7%)	2 (2.4%)	
Biliary		1 (2.3%)	10 (12%)	
Cryptogenic		5 (12%)	11 (13%)	
Other		3 (7.0%)	7 (8.5%)	
Viral Hepatitis		19 (44%)	21 (26%)	
Past Medical History				
Hypertension	128	16 (36%)	27 (32%)	0.63
BMI	121	27.49 (25.41, 31.30)	28.64 (25.32, 32.93)	0.70
Obesity	121			0.50
BMI <= 30		29 (66%)	46 (60%)	
BMI > 30		15 (34%)	31 (40%)	
**DONOR**				
Age (years)	128	49.00 (31.25, 60.00)	43.00 (29.75, 56.00)	0.27
Sex	128			0.95
Female		17 (39%)	32 (38%)	
Male		27 (61%)	52 (62%)	
Past Medical History				
Diabetes mellitus–insulin dependent	128	2 (4.5%)	3 (3.6%)	>0.99
Diabetes mellitus— Non-insulin dependent	128	4 (9.1%)	8 (9.5%)	>0.99
Hypertension	128	15 (34%)	36 (43%)	0.34

^1^ *n* (%). ^2^ Fisher’s exact test; Wilcoxon rank sum test; Pearson’s Chi-squared test.

**Table 2:** Top ten enriched pathways for all the nodes in the subnetwork 1, using metabolite-protein interaction from the KEGG database.

Pathway	Total	Hits	FDR
Glycerophospholipid metabolism	97	46	1.12 × 10^−70^
Ether lipid metabolism	47	34	8.65 × 10^−60^
Sphingolipid metabolism	47	30	1.08 × 10^−49^
alpha-Linolenic acid metabolism	25	21	2.08 × 10^−38^
Linoleic acid metabolism	29	21	5.51 × 10^−36^
EGFR tyrosine kinase inhibitor resistance	1490	65	6.78 × 10^−35^
Arachidonic acid metabolism	63	21	2.02 × 10^−26^
Alzheimer disease	41	14	1.91 × 10^−17^
Axon guidance	132	19	2.78 × 10^−16^
p53 signaling pathway	119	18	8.22 × 10^−16^

**Table 3:** Functional analysis of the modules identified with the InfoMap algorithm. FA = Functional Annotation; GO = Gene Ontology; BP = Biological Process; MF = Molecular Function; CC = Cellular Component.

Module	FA	Pathway	Hits (Range)/Total	FDR (Range)
0	GO:BP	cellular biogenic amine metabolic process	28/167	8.29 × 10^−43^
		glycerophospholipid metabolic process	32/327	8.29 × 10^−43^
		phospholipid metabolic process	34/463	3.36 × 10^−42^
		glycerophospholipid biosynthetic process	30/255	3.36 × 10^−42^
		phospholipid biosynthetic process	30/285	8.98 × 10^−41^
1	GO:BP	sphingolipid metabolic process	25/173	2.19 × 10^−41^
		membrane lipid metabolic process	25/233	2.98 × 10^−38^
		ceramide metabolic process	20/77	2.28 × 10^−37^
		phospholipid metabolic process	27/463	2.2 × 10^−35^
		sphingolipid biosynthetic process	19/80	1.39 × 10^−34^
0	GO:MF	phospholipase activity	29/112	2.61 × 10^−52^
		lipase activity	29/136	7.65 × 10^−50^
		phospholipase A2 activity	21/32	2.24 × 10^−47^
		hydrolase activity, acting on ester bonds	29/1010	5.71 × 10^−24^
		calcium ion binding	16/673	4.28 × 10^−10^
1	GO:MF	phosphoric ester hydrolase activity	13/496	2.09 × 10^−9^
		hydrolase activity, acting on ester bonds	13/1010	5.21 × 10^−6^
		N-acyltransferase activity	6/97	5.21 × 10^−6^
		phosphatase activity	8/373	6.21 × 10^−5^
		transferase activity, transferring acyl groups other than amino-acyl groups	6/193	1.83 × 10^−3^
0, 1	GO:CC	endoplasmic reticulum membrane	(13–14)/872	(1.6–7.1) × 10^−7^
		nuclear outer membrane–endoplasmic reticulum membrane network	(13–14)/894	(1.6–7.1) × 10^−7^
		endoplasmic reticulum part	(13–14)/1060	7.93 × 10^−7^–2.89 × 10^−6^
		endomembrane system	17/2160	9.65 × 10^−7^–1 × 10^−4^
		endoplasmic reticulum	(1417)/1660	(1.2–2.9) × 10^−6^

## Data Availability

Raw MS metabolomics data generated in this project is available as Supplementary Material (Supplementary Table S2). Deidentified clinical data for this project may be provided upon reasonable request to the corresponding author, subject to compliance with institutional ethical guidelines.

## Supplementary Materials

The following supporting information can be downloaded at: https://www.mdpi.com/article/10.3390/metabo15110700/s1, Figure S1: Graft survival probabilities were comparable between the treatment arms throughout the follow-up period; Table S1: PLS-DA 5-fold cross validation results; Table S2: Metabolic features with corresponding class, HMDB ID, and VIP score (Component 1); Table S3: Raw Data.

## Abbreviations

AA	amino acid
ALP	alkaline phosphatase
ALT	Alanine Aminotransferase
BA	bile acid
BSWH	Baylor Scott & White Health
BP	biological process
CC	cellular component
DAG	diacyl-glycerol
GO	Gene Ontology
HMDB	Human Metabolome Database
IQR	interquartile range
KEGG	Kyoto Encyclopedia of Genes and Genomes
LDL-C	low-density lipoprotein cholesterol
LT	Liver Transplantation
MELD	Model for End-Stage Liver Disease
MF	molecular function
mTORC1	mTOR complex 1
MASLD	Metabolic Dysfunction-Associated Steatotic Liver Disease
OLT	Orthotopic Liver Transplantation
PEMT	Phosphatidylethanolamine N-methyltransferase
PC	phosphatidylcholines
PLS-DA	Partial Least Square–Discriminant Analysis
SIR	sirolimus
S1P	sphingosine-1-phosphate
SCFAs	short-chain fatty acids
TAC	tacrolimus
VIP	Variable Importance in Projection.
