# Supplementary material for: Metabolomics Analysis Uncovers Distinct Profiles of Liver Post-Transplant Patients by Immunosuppression Regimen
Source: Metabolites. 2025 Oct 29;15(11):700. doi: 10.3390/metabo15110700 (PMC12654826; doi:10.3390/metabo15110700)
Supplement: Supplementary file 1 [file metabolites-15-00700-s001.zip › ST1.pdf]

Supplementary Table S1. PLS-DA 5-fold cross validation results.

MEASURE	1 COMPS	2 COMPS	3 COMPS	4 COMPS	5 COMPS
**ACCURACY**	0.71169	0.69662	0.72769	0.72738	0.712
**R2**	0.10922	0.42122	0.59302	0.69176	0.73865
**Q2**	0.049197	0.0319	0.1061	0.10515	0.084839
