# Supplementary figures and images for: Metabolomics Analysis Uncovers Distinct Profiles of Liver Post-Transplant Patients by Immunosuppression Regimen
Source: Metabolites. 2025 Oct 29;15(11):700. doi: 10.3390/metabo15110700 (PMC12654826; doi:10.3390/metabo15110700)

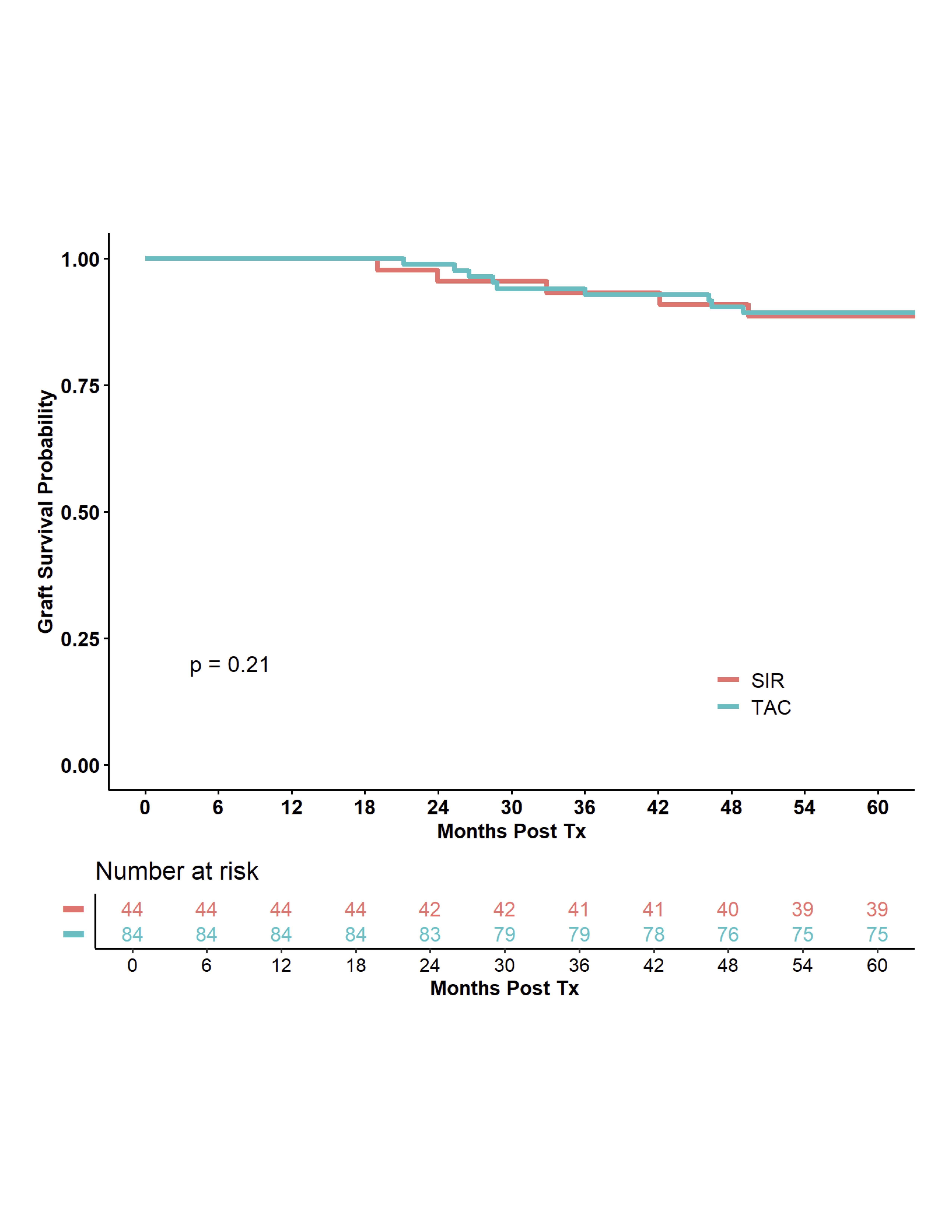

Supplement: Supplementary file 1 [file metabolites-15-00700-s001.zip › Sfig1.jpg]
